# Evaluation and management of *DMD* gene copy number variations detected by prenatal SNP-array testing

**DOI:** 10.1186/s12920-026-02333-6

**Published:** 2026-03-06

**Authors:** Jiancheng Hu, Jialun Pang, Rong Hu, Lin Zhou, Wenxian Yu, Hui Xi, Yingchun Luo, Shuting Yang, Wanglan Tang, Ai Hu, Jing Chen, Ying Peng

**Affiliations:** 1 Department of Medical Genetics, Hunan Provincial Maternal and Child Health Care Hospital, Changsha, China; 2 Department of Ultrasonography, Hunan Provincial Maternal and Child Health Care Hospital, Changsha, China

**Keywords:** Prenatal diagnosis, SNP-array, Copy number variations, Duchenne muscular dystrophy, Exon level variants

## Abstract

**Objective:**

This study aims to evaluate the clinical management of incidental findings of copy number variations (CNVs) in the DMD gene detected through prenatal single nucleotide polymorphism array (SNP-array).

**Methods:**

Prenatal SNP-array testing was performed on samples exhibiting CNVs in the DMD locus, followed by parental analysis using the same technique. Additionally, multiplex ligation-dependent probe amplification (MLPA) testing was conducted on prenatal cases and their parents. Pregnancy outcomes were documented, and postnatal follow-up was conducted.

**Results:**

SNP-array analysis identified copy number variations at Xp21 affecting either the entire DMD gene or only a portion of it in 14 fetuses. In 11 cases, MLPA testing confirmed the presence of deletions or duplications detected by the SNP-array.

**Conclusion:**

High-density SNP-array platforms with low reporting thresholds may incidentally detect a subset of exon-level copy number variations involving the DMD gene during routine prenatal testing and thereby contribute to early recognition of potential dystrophinopathy-related variants. Suspected DMD-related CNVs, especially exon-level alterations, require confirmation by targeted assays such as MLPA or next-generation sequencing, together with cautious clinical interpretation. Assessing the pathogenicity of prenatally detected DMD copy number variations remains challenging, particularly for duplications, which require careful evaluation. Furthermore, the sequential, time-intensive nature of confirmatory and familial studies often limits definitive risk assessment within the prenatal decision-making window, and introduces broader familial implications that must be navigated through careful, multidisciplinary counseling.

**Supplementary Information:**

The online version contains supplementary material available at 10.1186/s12920-026-02333-6.

## Introduction

Duchenne muscular dystrophy (DMD; OMIM #310200), Becker muscular dystrophy (BMD; OMIM #300376), and X-linked DMD-associated dilated cardiomyopathy (DCM; OMIM #302045) are X-linked recessive neuromuscular disorders caused by pathogenic variants in the dystrophin gene. DMD patients commonly exhibit early symptoms such as elevated creatine kinase levels or exercise intolerance, typically emerging at an average age of 3 ± 1.8 years. In contrast, BMD presents with greater variability, with symptom onset occurring at an average age of 12.9 ± 11.8 years. The condition is characterized primarily by elevated creatine kinase levels and symptoms such as muscle weakness, fatigue, myalgia, or cramping [1]. DMD patients typically exhibit severe, often life-threatening symptoms, including progressive and irreversible muscle weakness and atrophy, frequently accompanied by pseudohypertrophy of the gastrocnemius muscle. Most individuals with DMD succumb to respiratory or cardiac failure in their third or fourth decade of life [2, 3]. The dystrophin gene, consisting of 79 exons, is one of the largest protein-coding genes in the human genome, spanning approximately 2.4 Mb [4]. The dystrophin protein, encoded by the DMD gene, consists of 3,686 amino acids. Mutations in the DMD gene impair dystrophin function, disrupting the linkage between the actin cytoskeleton and the extracellular matrix. Consequently, muscle fibers become vulnerable to contraction-induced damage, resulting in chronic muscle degeneration, inflammation, and progressive functional impairment [5]. Pathogenic alterations in the DMD gene include large rearrangements and point mutations. Deletion and duplication of intact exons constitute the primary pathogenic mechanisms of DMD/BMD. Deletions account for approximately 60–70% of cases, whereas duplication in the DMD gene comprise approximately 5–20% [4, 6–10].

Although single nucleotide polymorphism array (SNP-array) is not the primary diagnostic tool for DMD due to its limited sensitivity in detecting exon-level deletions and duplications, CNVs in this region are sometimes incidentally identified during prenatal diagnosis using SNP-array.

## Materials and methods

### Subjects

This study included 9,807 fetuses who underwent prenatal diagnosis using SNP-array technology between January 2019 and March 2023 at the Prenatal Diagnosis Center. The indications for prenatal diagnosis were based on high-risk factors, including advanced maternal age (≥ 35 years), abnormal non-invasive prenatal testing (NIPT) results, abnormal fetal ultrasound findings (structural anomalies or soft markers), parental chromosomal abnormalities, or a history of adverse pregnancy outcomes. Among them, 14 fetuses from 13 families exhibited copy number variations within the DMD region during prenatal diagnosis (Table 1). This study was approved by the Ethics Committee of Hunan Provincial Maternal and Child Health Hospital. Informed consent was obtained from the patient or their legal guardian to ensure adherence to ethical standards.

**Table 1:** Information on 14 fetuses (including *DMD* region copy number variation) and the results of karyotyping, SNP analysis and MLPA

Case No.	Mother age	Gestation	Fetus age	Sample Type	Indication for prenatal diagnosis	Fetus sex	Karyotype	SNP-array results				MLPA results	ParentalOrigin	Genetic diagnosis	Pathogenicity rating	Pregnancy outcome
								(GRCh37) start–end	size (kb)	gene	Type					
1	32 Y	G2P0	21 W	Amniotic fluid	High Risk: NIPT Chromosome 14	M	46, XY	Xp21.1(32766676_33051418) x0	285	Exon 2–7	del	Del exon 2–7	maternal	Del exon 2–7	P	Termination of pregnancy
2	34 Y	G1P2	28 W	Amniotic fluid	Ultrasound: fetal ventricular septal defect	M	46, XY	Xp21.1(31776453_32032326) x0	256	Exon 45–51	del	Del exon 45–51	maternal	Del exon 45–51	P	Termination of pregnancy
3	20 Y	G2P0	25 + W	Amniotic fluid	Ultrasound: fetal ventricular septal defect	M	46, XY	Xp21.1(32,547,461_33,916,403) x2	1 360	Exon 1–17	dup	Dup exon 1–20	maternal	Dup exon 1–20	VUS	Termination of pregnancy
4	21 Y	G3P1	20 W	Amniotic fluid	Maternal clinical features (mild intellectual disability, dysarthria, gait instability) and history of adverse pregnancy outcome.	M	46, XY	Xp21.1(32506756_33943923) x2	1 440	Exon 1–20	dup	N/A	maternal	Dup exon 1–20	VUS	Termination of pregnancy
5	28 Y	G2P1	22 W	Amniotic fluid	Previous Child with DMD	M	46, XY	Xp21.1(32498836_32578660) x2, Xp21.1(31889200_32216814) x2, Xp21.1(31614608_31658813) x2	80, 328, 44	Exon 17–21, 45–47, 55,	dup	Dup exon 17–21, 45–48, 55	maternal	Dup exon 17–21, 45–48, 55	P	Termination of pregnancy
6	33 Y	G2P1	31 + W	Amniotic fluid	Ultrasound: fetal ventricular septal defect	M	46, XY	Xp21.2p21.1(31385522_34518624) x2	3 130	Exon 1–60	dup	Dup exon 1–60	maternal	Dup exon 1–60	VUS	Stillbirth
7	34 Y	G5P2	16 W	Amniotic fluid	Abortion of previous fetus with Klinefelter syndrome	M	46, XY	Xp21.2(30947295_31383813) x2	437	Exon 61–79	dup	Dup exon 61–79	maternal	Dup exon 61–79	VUS	Born, Healthy
8	23 Y	G3P2	21 W	Amniotic fluid	Stillbirth of one twin in this pregnancy	M	46, XY	Xp21.2(30415814_31186578) x2	771	Exon 75–79	dup	Dup exon 74–79	maternal	Dup exon 74–79	VUS	Born, Healthy
9	28 Y	G2P1	17 W	Amniotic fluid	NIPT: High risk of Sex Chromosome aneuploidy	F	46,X, del(X)(p21p22)	Xp22.11p21.1(22,421,082_32,898,332) x1	10 500	DMD Full-length	del	N/A	Unavailable	46,X, del(X)(p21p22)	P	Termination of pregnancy
10	26 Y	G3P1	16 + W	Amniotic fluid	Previous Child with DMD	F	46, XX	Xp21.1(31,671,937_31,872,842) x1	200	Exon 49–53	del	Del exon 49–54	maternal	Del exon 49–54	P	Born, Healthy
11	31 Y	G2P1	17 W	Amniotic fluid	High risk of Down syndrome	F	46, XX	Xp21.1(32491564_32544026) x1	52	Exon 18–22	del	N/A	Unavailable	Del exon 18–22	P	No Amniotic fluid, Termination of pregnancy
12	31 Y	G3P1	24 W	Amniotic fluid	Ultrasound: right aortic arch with vascular ring, right clubfoot, and mild left renal pelvic dilatation.	F	46, XX	Xp21.1(32931546_33685657) x3	754	Exon 1–2	dup	Dup exon 1–2	Unavailable	Dup exon 1–2	VUS	Ultrasound Abnormalities, Termination of pregnancy
13	36 Y	G6P2	16 + W	Amniotic fluid	High risk NIPT	F	46, XX	Xp21.1(33112142_33623734) x3	512	Exon 1	dup	Dup exon 1	paternal	Dup exon 1	LB	Born, Healthy
14	34 Y	G2P1	16 W	Amniotic fluid	High risk of Down syndrome	F	46, XX	Xp21.1(32561825_32614364) x3	53	Exon 13–17	dup	Normal		Normal	N/A	Born, Healthy

Fetuses 3 and 4 are two fetuses of the same woman. *P* Pathogenic, *VUS* Variant of Uncertain Significance, *LB* Likely Benign, *F* Female, M Male, *N/A* Not detected or not rated, Unavailable, No pedigree testing was done and the source could not be determined

### Karyotyping, single nucleotide polymorphism array analyses, and multiplex ligation-dependent probe amplification (MLPA) analysis

#### G-band karyotype

Analysis was performed on fetal amniotic fluid and peripheral blood samples obtained from the parents. A series of analytical procedures was conducted on the samples, including inoculation, culturing, hypotonic treatment, fixation, imaging, and Giemsa staining. Subsequently, the G-banded karyotype of metaphase chromosomes was analyzed.

#### SNP-array assay

Genomic DNA was extracted from uncultured amniotic fluid cells using the QIAamp DNA Mini Kit (Qiagen, Germany, Cat. No. 51304), following the manufacturer’s protocol for ‘DNA Purification from Blood or Body Fluids (Spin Protocol)’. The CytoScan 750 K SNP-array, manufactured by Affymetrix (USA), was used. This array includes approximately 200,000 SNP probes and 550,000 chromosomal copy number variants (CNVs). A total of 250 ng genomic DNA was used, following the experimental procedures outlined in the manual. The Affymetrix Fluidics Station 450Dx was used for washing and scanning with the Affymetrix 7G system, followed by signal analysis using Affymetrix CHAS software.

#### MLPA analysis

MLPA analysis was performed using the P034 and P035 kits from MRC-Holland (Amsterdam, Netherlands). Each sample was tested with 5 µL of DNA. The procedure involved denaturation, hybridization, ligation, and PCR. The amplified products were separated using ABI 3500 sequencer capillary electrophoresis, and the results were analyzed with Coffalyser.NET. A 30% increase or decrease in the relative peak area indicated exon duplications or deletions in the DMD gene.

#### Variant classification

Genomic coordinates and gene content of detected CNVs were obtained from the UCSC Genome Browser (http://genome.ucsc.edu/). Previously reported DMD variants were queried in the Human Gene Mutation Database (HGMD) and the Leiden Open Variation Database (LOVD; http://www.lovd.nl/). Exon-skipping–related information was obtained from databases developed by Leiden University Medical Center (https://www.dmd.nl/) and CureDuchenne (https://cureduchenne.org/exon-skipping/).

All variants were interpreted and classified according to the ACMG-AMP guidelines and ACMG/ClinGen technical standards for CNV interpretation [11, 12]. Variants were categorized as pathogenic, likely pathogenic, variants of uncertain significance (VUS), likely benign, or benign based on genomic content, predicted impact on the DMD reading frame, inheritance pattern, segregation analysis, and available clinical information. For duplications and other CNVs with uncertain clinical significance, DMD-specific evidence and reported genotype–phenotype correlations were additionally considered. When evidence was conflicting or insufficient, variants were conservatively classified as VUS.

#### Postnatal follow-up and outcome assessment

Postnatal follow-up was conducted for all live-born infants through a combination of medical record review and direct contact with families via outpatient visits or telephone interviews. The minimal follow-up duration was 6 months after delivery, and extended follow-up was available for selected cases. Clinical endpoints collected during follow-up included growth and developmental milestones, neuromuscular symptoms, motor performance, and serum creatine kinase (CK) levels when available. For cases with pregnancy termination, clinical indications and prenatal phenotypic findings were extracted from medical records. Follow-up data were used to support postnatal phenotype correlation and variant reclassification when necessary.

## Results

**Fig. 1:**
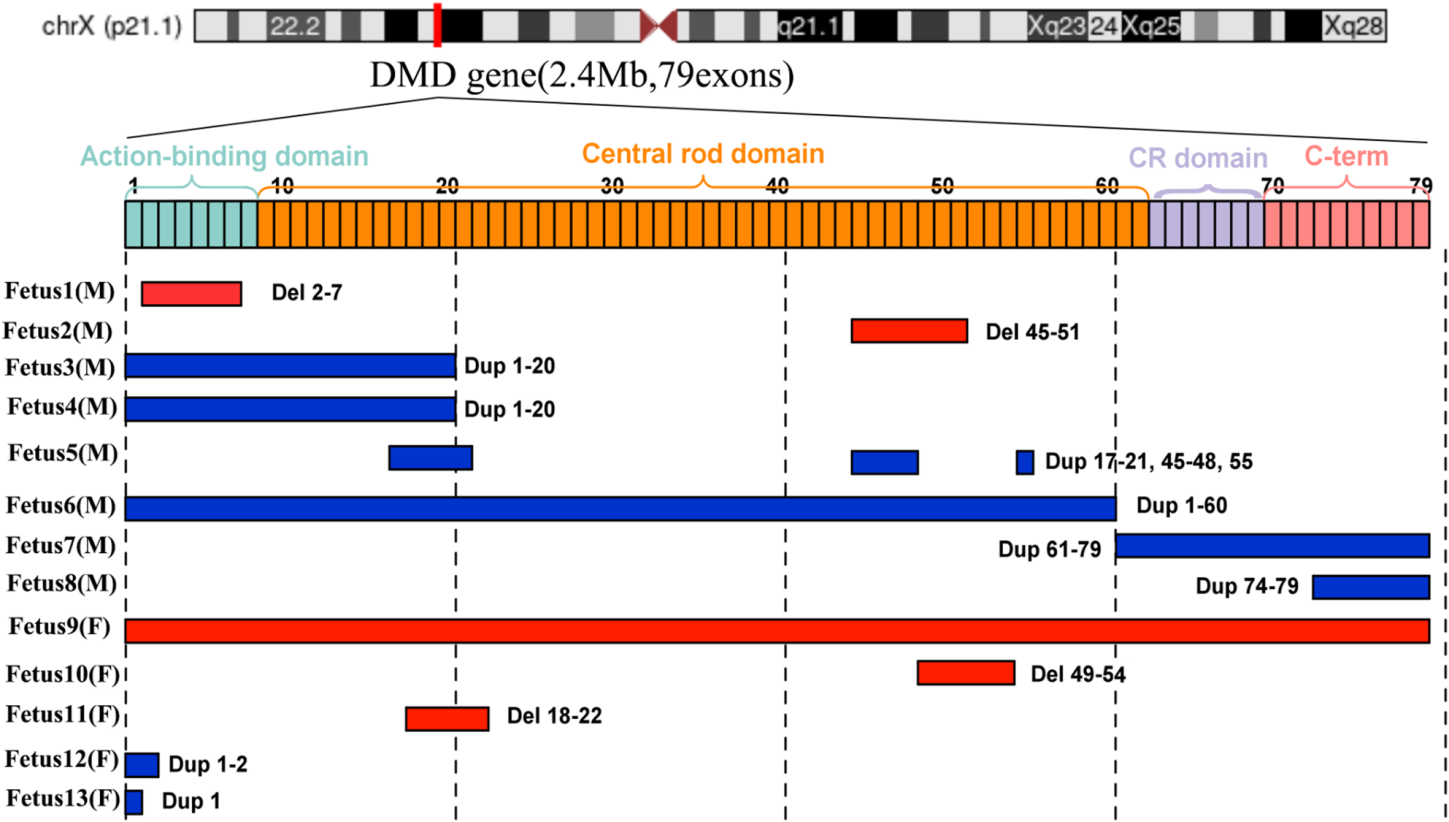
Exon distribution of DMD copy number variations detected prenatally. The DMD gene structure (2.4 Mb, 79 exons) and dystrophin functional domains are shown at the top. Colored bars indicate exon-level copy number changes observed in each fetus. Red indicates deletions and blue indicates duplications (copy number gains). Fetal sex is indicated as male (M) or female (F)

G-band karyotype analysis was performed on 14 fetuses, revealing normal karyotypes in all cases except for fetus 9, which exhibited a notable deletion in the Xp21p22 locus, encompassing the DMD gene. Copy number variation of Xp21 (overlapping the DMD gene) was identified in all 14 fetuses through SNP-array analysis. Following this, twelve fetuses, along with their corresponding families, underwent further testing using MLPA. The specific results are shown in Table 1; Fig. 1. The SNP-array pinpointed minimal deletion and duplication sizes of 52 kb and 44 kb, respectively. MLPA analysis confirmed that the location and size of the duplications or deletions were consistent in 11 fetuses identified by SNP-array. For fetus 14, the SNP-array indicated a 53 kb duplication, inclusive of DMD gene exons 13–17; however, the MLPA assay did not subsequently support the array result. Fetus 9 harbored a deletion that spanned the entire DMD region, while the other fetuses exhibited deletions or duplications involving only sections of the DMD gene.

Parental analysis using SNP-array or MLPA analysis revealed maternal origins for the copy number variants in nine fetuses, while only the variant in fetus 13 was paternal in origin. Three non-consecutive segment duplications were identified in fetus 5, namely in exons 17–21, 45–48, and exon 55. The maternal grandfather of fetus 7 carries a duplicated fragment at the same position and size, without any clinical symptoms. This suggests that the presence of a copy number fragment containing exons 61–79 of the DMD gene might not impede the functionality of dystrophin and does not predispose to the development of the disease. Postnatal follow-up confirmed the robust health of fetus 7. Fetus 8 exhibits a hemizygous duplication involving exons 74–79 of the DMD gene. This trait is inherited maternally. Regrettably, the sample from his maternal grandfather could not be obtained. Fetus 8 remained in good health after delivery. During postnatal follow-up, families of fetuses 7 and 8 reported that postnatal serum creatine kinase (CK) testing yielded normal results, and no neuromuscular abnormalities were observed. Fetuses 3 and 4 represent two gestations of the same woman. The mother, who carries the duplication, presented with a clinical history of mild cognitive deficits, speech impairments, and gait instability. Given the familial history and the presence of the same duplication in the male fetuses, alongside other obstetric considerations, the family opted for pregnancy termination after comprehensive genetic counseling.

Fetuses 9–14 were identified as female carriers. In fetus 9, the deleted fragment was located in Xp22.11p21.1, encompassing a heterozygous deletion of approximately 10.5 Mb, which involves 36 protein-coding genes. According to the ACMG guidelines, the deleted fragment is categorized as pathogenic, which led the parents to choose pregnancy termination. The pregnancy of fetus 11 was terminated due to anhydramnios, and the pregnancy of fetus 12 was terminated due to structural anomalies, with the DMD copy number variations being unlikely to be the causative factor. Fetuses 10, 13, and 14 were successfully delivered and exhibited good health during subsequent follow-up assessments.

## Discussions

This study focuses on incidentally detected DMD copy number variations identified by genome-wide prenatal SNP-array screening in an unselected fetal cohort. By integrating parental segregation analysis, MLPA confirmation, and postnatal follow-up, our series extends current prenatal DMD literature beyond family-based targeted testing and provides practical insights into CNV-centered interpretation and counseling challenges.

### Performance of prenatal SNP-array and MLPA in incidental DMD CNV detection

Traditionally, prenatal diagnosis of Duchenne muscular dystrophy is performed in families with an established proband or confirmed carrier status, requiring prior identification of the pathogenic DMD variant and correlation with the proband’s clinical phenotype. In such settings, MLPA is one of the most widely used techniques, enabling rapid and accurate detection of large exon-level deletions and duplications, whereas sequence-based methods are primarily applied to identify small intragenic insertions/deletions as well as missense, nonsense, and splice-site variants. Subsequently, carrier testing of the mother is required to confirm the presence of the same pathogenic variant, followed by appropriate timing of invasive prenatal sampling and targeted molecular testing of fetal DNA. In contrast to this conventional targeted diagnostic workflow, the present study focuses on incidentally detected DMD copy number variations identified through genome-wide prenatal SNP-array screening in an unselected fetal cohort. By systematically characterizing these unexpected CNV findings, integrating parental segregation analysis, MLPA confirmation, and postnatal outcome follow-up, this series provides practical evidence for the clinical interpretation and counseling challenges associated with CNV-centric prenatal detection of DMD-related variants, thereby extending current knowledge beyond traditional family-based prenatal diagnostic paradigms.

MLPA is a reliable and expeditious method capable of detecting the complete *DMD* gene and precisely determining the presence of deletions or duplications in specific exons [13, 14]. However, it is pertinent to note that the clinical manifestations of muscular dystrophy do not present during the fetal developmental phase, which may result in reluctance among prospective mothers without a genetic predisposition to undergo MLPA testing for the DMD gene. This study examined 9,807 fetuses who received prenatal diagnoses via SNP-array technology from January 2019 to March 2023. Of these, 14 fetuses displayed copy number variations (CNVs) in the *DMD* gene region. When CNVs are unexpectedly detected during prenatal diagnosis, a comprehensive review of the family history of the pregnant woman is recommended. Genetic testing of the parents and other pertinent family members can facilitate the early detection of dystrophinopathies and mitigate the associated risks. The resolution and accuracy of the SNP-array are contingent upon the probe density on the chip and the configuration of the test sites. The microarray employed in this study featured 750,000 probes in total, including 200,000 SNP probes and 550,000 CNV probes, uniformly distributed across the entire human genome at an average interval of approximately one probe every 4 kb. For instance, in fetus 5, the array was proficient in detecting segmental gains of 80 kb, 328 kb, and 44 kb in size, corresponding to duplications spanning exons 17–21, exon 45–47, and exon 55, respectively. The presence of these duplications was corroborated by MLPA confirmation. Taken together, these findings indicate that high-density SNP-array platforms may incidentally detect a subset of exon-level DMD copy number variations during routine prenatal screening and thereby contribute to early recognition of potential dystrophinopathy-related variants. However, SNP-array should not be considered a primary diagnostic tool for DMD, particularly given its limited sensitivity for single-exon events, mosaicism, and breakpoint resolution. All suspected DMD-related CNVs identified by microarray require confirmation by targeted assays such as MLPA or next-generation sequencing, together with cautious clinical interpretation.

### Potential pitfalls and confirmation necessity

However, it is crucial to acknowledge the limitations of SNP-array in this context. In our cohort, one case (Fetus 14) presented a discrepancy where a 53 kb duplication (exons 13–17) was indicated by SNP-array but not confirmed by MLPA. Potential sources of such false-positive signals include technical artifacts due to local genomic complexity (e.g., segmental duplications), suboptimal probe performance, or low-level mosaicism. This case underscores a critical principle: SNP-array serves as a screening, not a diagnostic, tool for DMD CNVs. Any suspected DMD-related CNV, particularly duplications, requires mandatory confirmation by orthogonal, targeted methods such as MLPA or next-generation sequencing before informing clinical decisions.

### Proposed management pathway for incidental findings

Based on our experience, we propose a structured approach for managing incidental DMD CNVs detected by prenatal SNP-array:

1. Confirmation and Refinement: Confirm the CNV with a targeted assay (MLPA) and perform parental segregation analysis to determine inheritance.
2. Familial Phenotypic Correlation: Obtain a detailed family history and, if possible, test relevant relatives to correlate the variant with any historical or present symptoms of dystrophinopathy.
3. Integrated Risk Assessment: Combine genetic data (variant type [deletion/duplication], reading frame effect, inheritance) with fetal sex to assess pathogenicity. For male fetuses, frame-disrupting deletions are high-risk for DMD, while in-frame changes may suggest BMD. For female fetuses, carrier status is the primary concern, with a rare risk of symptomatic disease.
4. Multidisciplinary Counseling and Decision-making: Provide comprehensive genetic counseling involving clinical geneticists, maternal-fetal medicine specialists, and pediatric neurologists to discuss uncertain results, phenotypic variability, and reproductive options.
5. Postnatal Planning: For pregnancies continued, establish a plan for postnatal evaluation (e.g., serum creatine kinase measurement, clinical neuromuscular assessment) to enable early diagnosis and management if symptoms emerge.

### Interpretation challenges of DMD duplications

The pathogenicity and severity of variants in the *DMD* gene hinge on whether they disrupt the structure of the open reading frame at the site of mutation. In instances of significant deletions or duplications in the *DMD* gene, or point mutations that interrupt or alter the mRNA-encoded reading frame, the production of dystrophin is either obstructed or rendered inoperative. Consequently, patients typically present with severe symptoms of DMD. Sequence or copy number variants allowing the mRNA-encoding reading frame to remain intact, yet leading to the partial loss or duplication of dystrophin, allowing for partial expression and functionality of dystrophin, typically result in a less severe clinical phenotype of Becker muscular dystrophy, with some exceptions [15–19]. Data gathered from the Leiden Muscular Dystrophy Database (LMDp) (www.dmd.nl) validate that duplications primarily originate from the paternal allele in the majority of cases. The reading frame rule is typically applied to repetitions under the assumption that they occur in tandem. However, it has been found that this assumption is incorrect in cases where the concatenated repetitions may actually involve transposition. This distinction is crucial since translocations do not affect the reading frame, especially when occurring outside of the *DMD* gene [9]. For accurate clinical diagnosis, it is essential to ascertain whether duplications conform to the reading frame rule and warrant further analysis, particularly in cases where the clinical phenotype does not align with the genotype, necessitating breakpoint analysis [20]. Duplications occurring outside the *DMD* gene typically do not alter the gene’s reading frame nor lead to its inactivation. Given that the protein encoded by the *DMD* gene remains almost intact without grave consequences, caution is advised when predicting phenotypes purely based on genotypic information.

### Genetic counseling considerations for female carriers

BMD/DMD is an X-linked recessive disorder that typically remains asymptomatic in female carriers. A probable explanation suggests that for a female fetus harboring a single X chromosome with a copy number variant, health is generally unaffected post-birth due to the inactivation of the affected X chromosome. In this study, fetuses 9–13 were all female carriers. Fetuses 10 and 13 were successfully delivered and subsequent follow-up assessments confirmed their good health. In rare cases, DMD can manifest in females, especially when a balanced X-autosome reciprocal translocation disrupts the *DMD* gene, coinciding with skewed X-inactivation. Consequently, females with such translocations are incapable of synthesizing dystrophin. To date, the literature has documented 26 cases of balanced translocation involving the *DMD* gene, resulting in the manifestation of the DMD phenotype in females. Female carriers exhibiting symptoms of DMD show skewed X-chromosome inactivation, wherein the X chromosomes with normal alleles are predominantly inactivated. Conversely, asymptomatic carriers and individuals in the control group display random X-chromosome inactivation [21, 22]. The presentation of symptoms in females may correlate with the levels of dystrophin expression. The occurrence of severe symptoms in females is associated with a range of genetic mechanisms, including notable deletions at Xp21.2, X-chromosome rearrangements at Xp21.2, 45X [23], uniparental disomy (UPD) of the X chromosome [24], the presence of two pathogenic variants in DMD, and non-random X chromosome inactivation [25, 26]. Therefore, the phenotypic correlation for prenatal prediction of *DMD* variants in females is intricate. It is important to note that a significant proportion (15–20%) of female carriers may develop symptoms, ranging from mild muscle weakness to severe, DMD-like manifestations, including cardiomyopathy [27]. Consequently, current clinical guidelines recommend that all identified female carriers undergo lifelong, regular cardiac surveillance (e.g., echocardiography and/or cardiac MRI) regardless of symptomatic status, to enable early detection and management of cardiomyopathy [28]. This information is a critical component of post-test genetic counseling.

### Clinical and practical implications for prenatal management

Approximately two-thirds of DMD cases stem from mutations inherited from carrier mothers [29]. In this study, all copy number variants observed in the eight male fetuses were all derived from their respective mothers. Noteworthy is that variants 1, 2, and 5 in the fetuses were classified as pathogenic and resulted in the termination of these pregnancies. Fetuses 3 and 4 represent two gestations of a same woman. The family chose to proceed with labor induction with full informed consent, due to the fetuses’ mild cognitive deficits, speech impairments, unstable gait, and identical genetic sequence repetitions. Fetus 7 harbored a hemizygous duplication within *DMD* gene spanning exons 61–79, mirroring a genetic anomaly also found in the fetus’s grandfather. Fetus 8 exhibited a hemizygous copy number gain in exons 74–79 of the *DMD* gene. The duplication was absent in the maternal uncles and grandmother of fetus 8. Variants present in Fetuses 7 and 8 were classified as uncertain significance during prenatal testing but the abnormalities in fetuses 7 and 8 were later deemed benign postnatally. The nonspecific nature of muscular dystrophy symptoms arising from *DMD* mutations complicates their detection in utero. Accurately assessing the pathogenicity of detected *DMD* gene copy number variant fragments present significant challenges in prenatal diagnosis. Owing to the difficulties in identifying relevant phenotypic traits, any potential incidence of fetal BMD or DMD or DCM can only be hypothesized through extensive familial testing. This process involves determining if the condition originates from a new mutation, pinpointing affected family members, consulting a range of databases and literature, and predicting the implicated exons.

A significant practical challenge in managing incidental DMD CNVs is the timeline for comprehensive evaluation. The stepwise process of confirmatory testing (MLPA), parental segregation analysis, and potentially expanded familial studies to establish phenotypic correlation can be time-consuming. In many cases, these investigations may not be completed within the typical window for considering pregnancy termination, adding considerable complexity to prenatal counseling and decision-making. Furthermore, access to extended family members for testing may be limited by geographical, social, or personal factors. In healthcare systems without universal coverage, the costs of cascade testing for family members may also pose a barrier. These logistical and ethical constraints must be openly discussed during genetic counseling. Importantly, this process can have secondary implications, such as identifying presymptomatic at-risk relatives (e.g., maternal aunts, cousins) who may benefit from surveillance, which carries both potential health benefits and psychosocial impacts.

## Supplementary Information

Supplementary Material 1.

## Data Availability

The datasets generated and analyzed during the current study are available in the ClinVar repository, https://www.ncbi.nlm.nih.gov/clinvar/, under the accession numbers Fetus1 (SCV006550912), Fetus2 (SCV006550919), Fetus3 (SCV006550920), Fetus4 (SCV006550921), Fetus5 (SCV006550922, SCV006550923, SCV006550924), Fetus6 (SCV006550925), Fetus7 (SCV006550926), Fetus8 (SCV006550913), Fetus9 (SCV006550914), Fetus10 (SCV006550915), Fetus11 (SCV006550916), Fetus12 (SCV006550917), Fetus13 (SCV006550918).
