# Supplementary figures and images for: Evaluation and management of DMD gene copy number variations detected by prenatal SNP-array testing
Source: BMC Med Genomics. 2026 Mar 6;19:61. doi: 10.1186/s12920-026-02333-6 (PMC13088639; doi:10.1186/s12920-026-02333-6)

# Result of MLPA

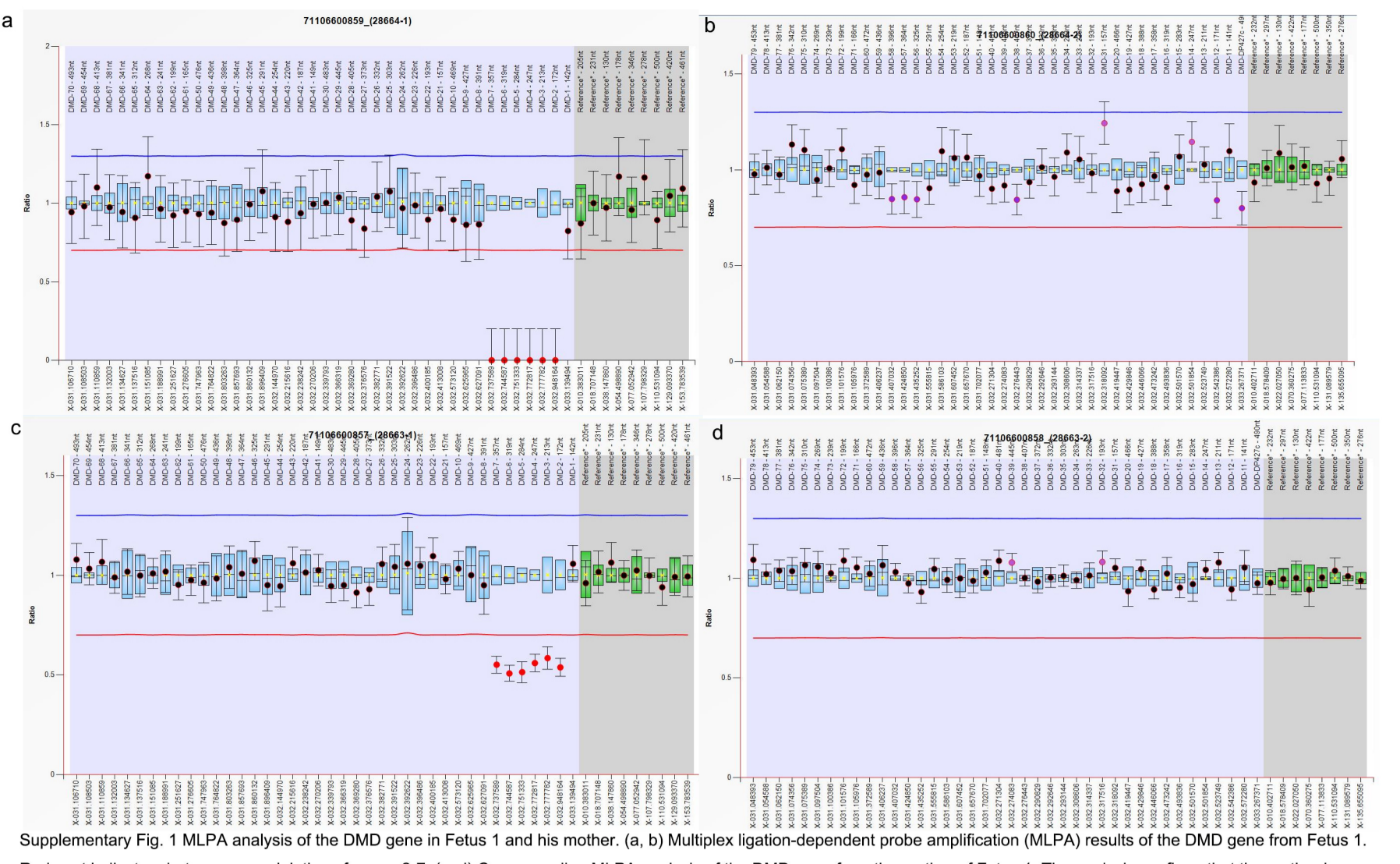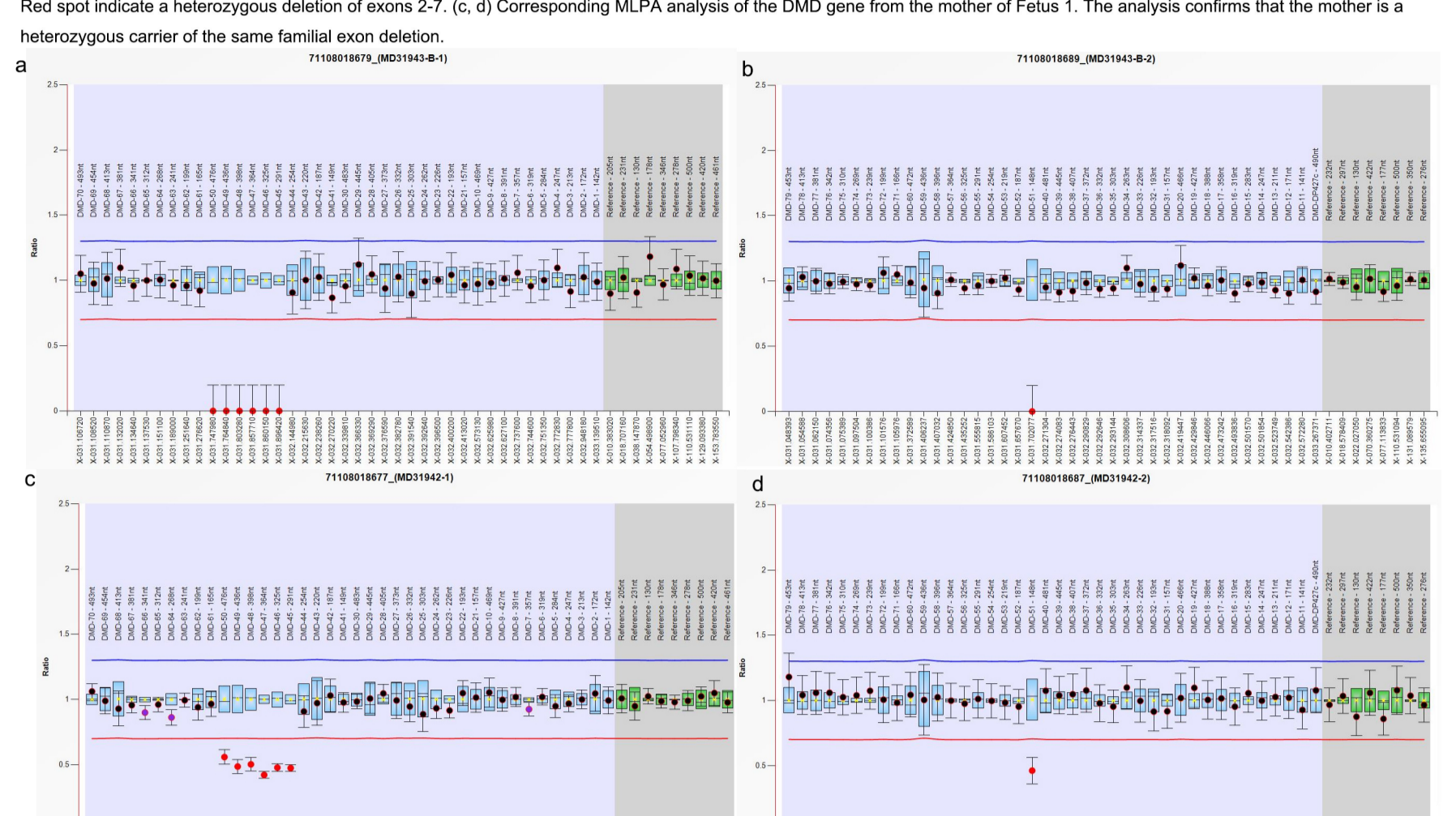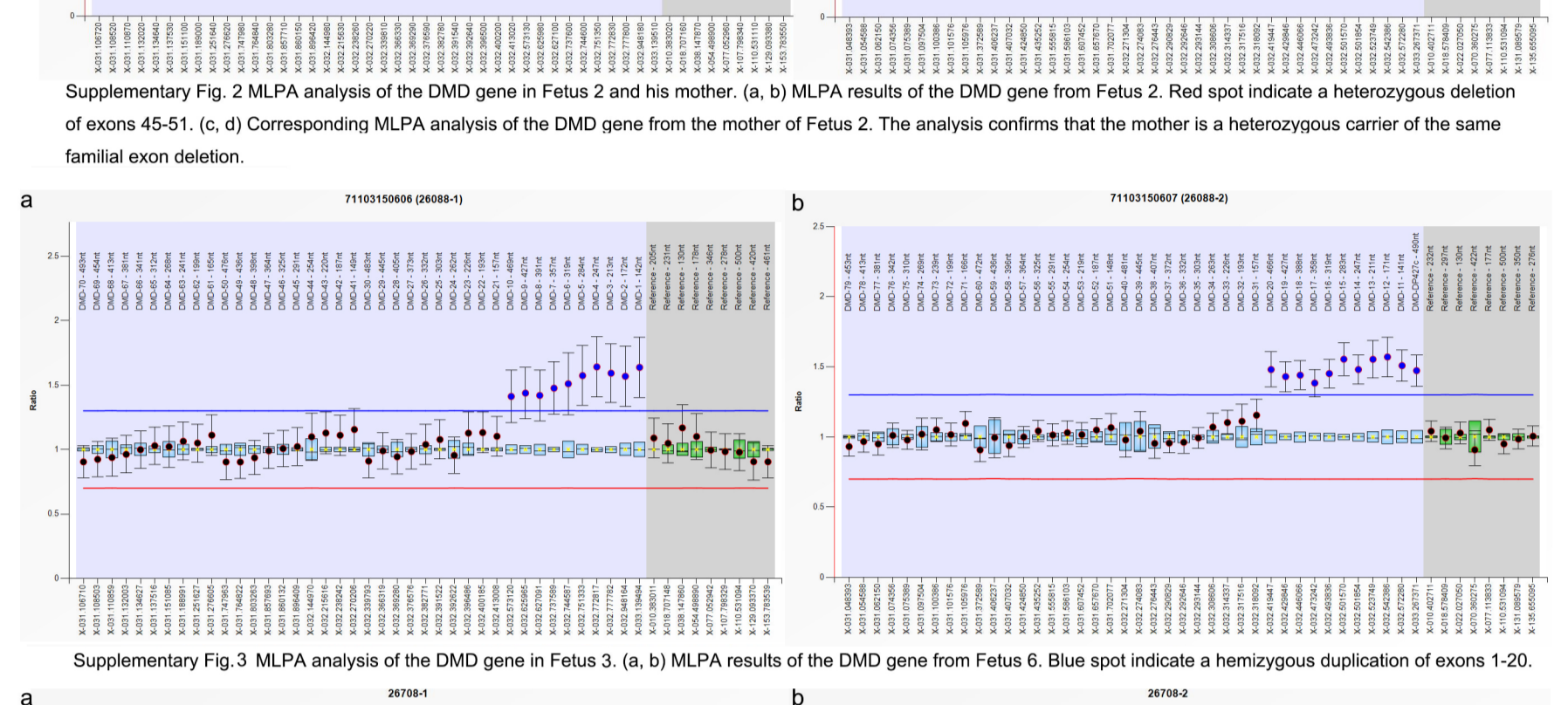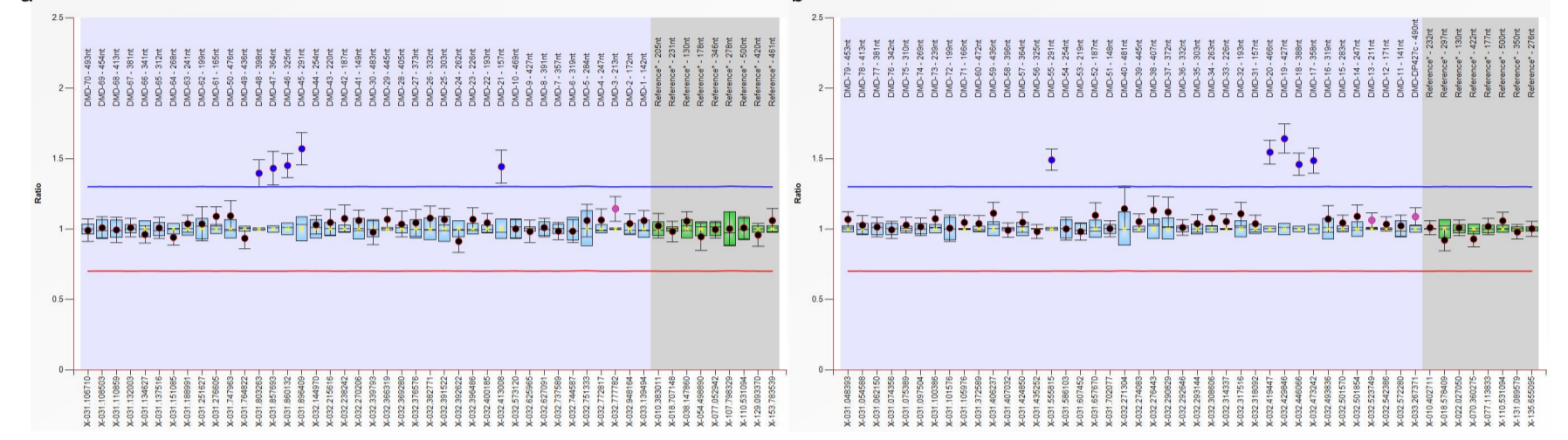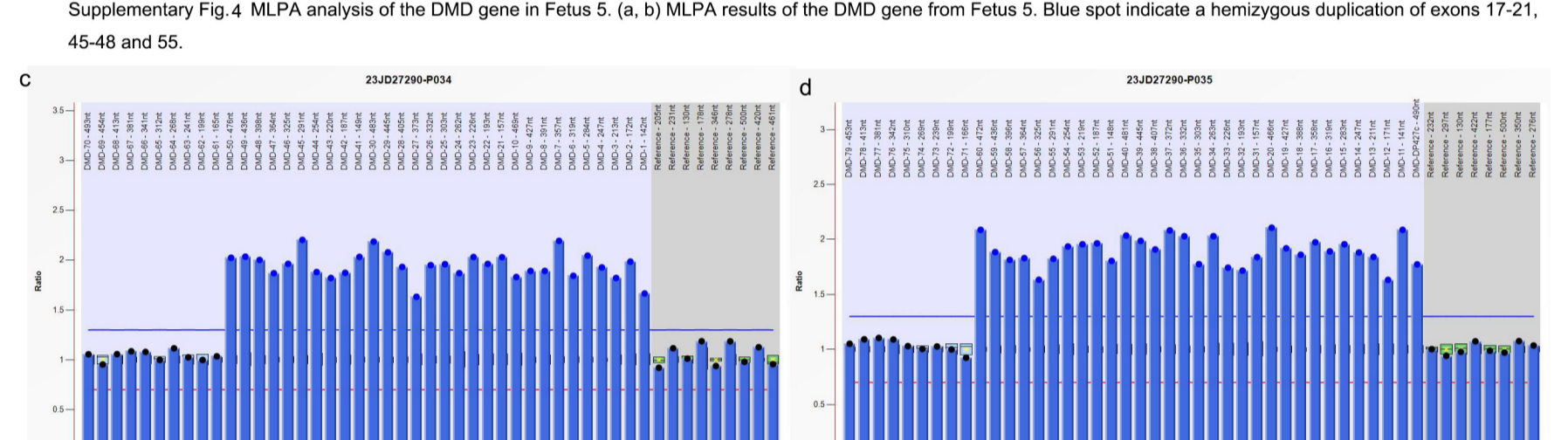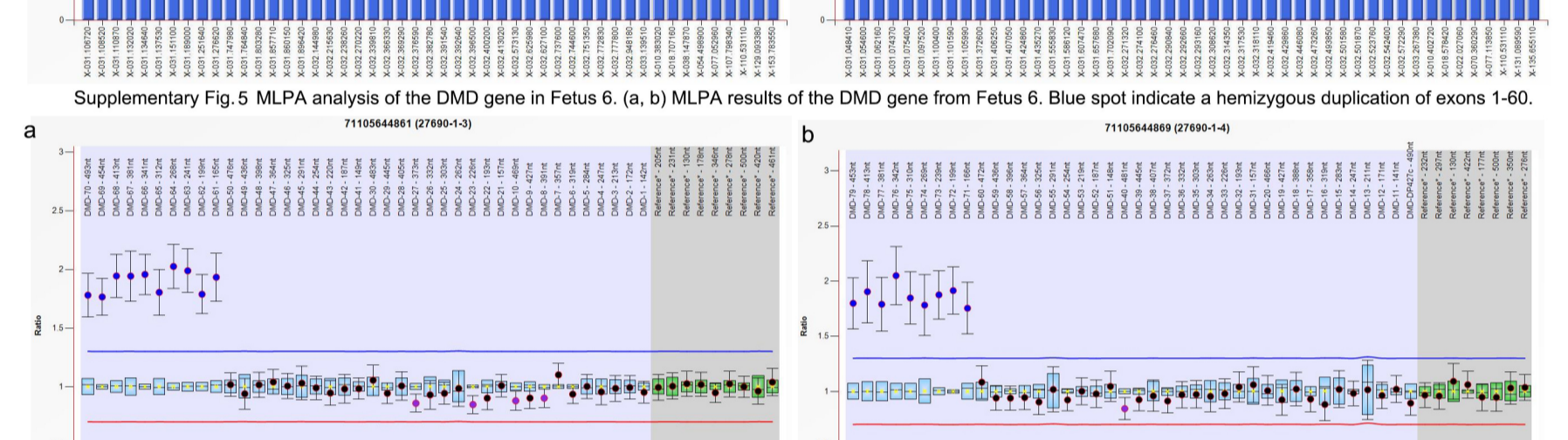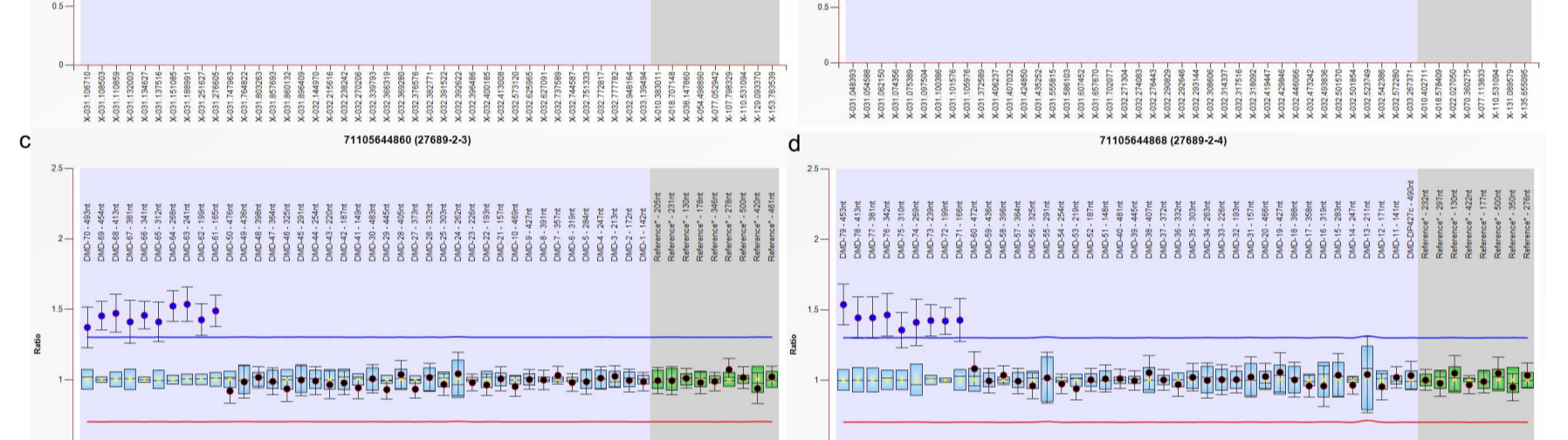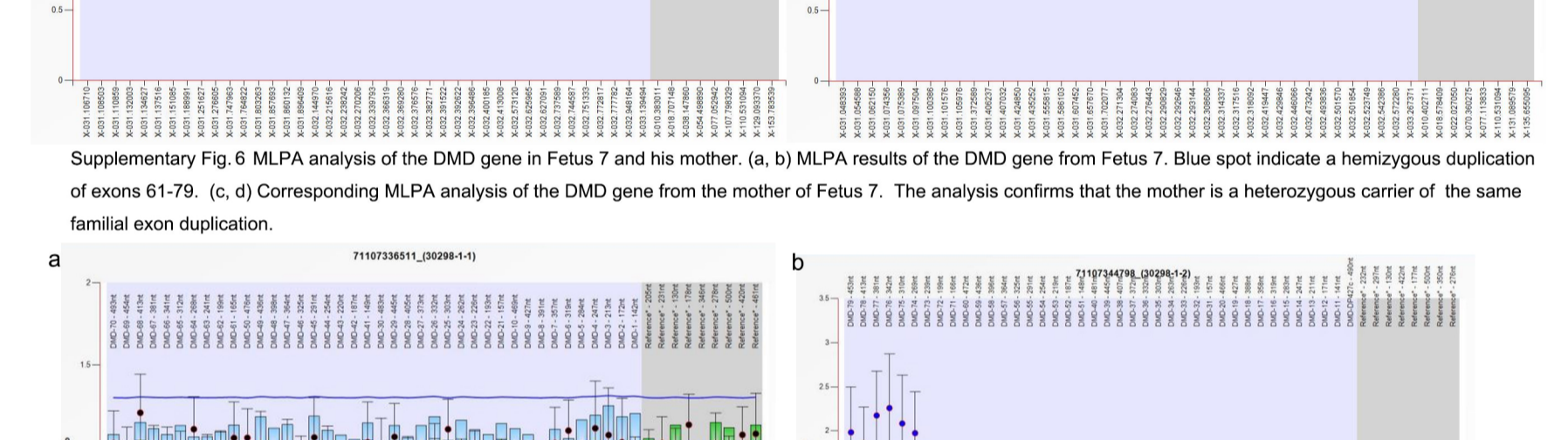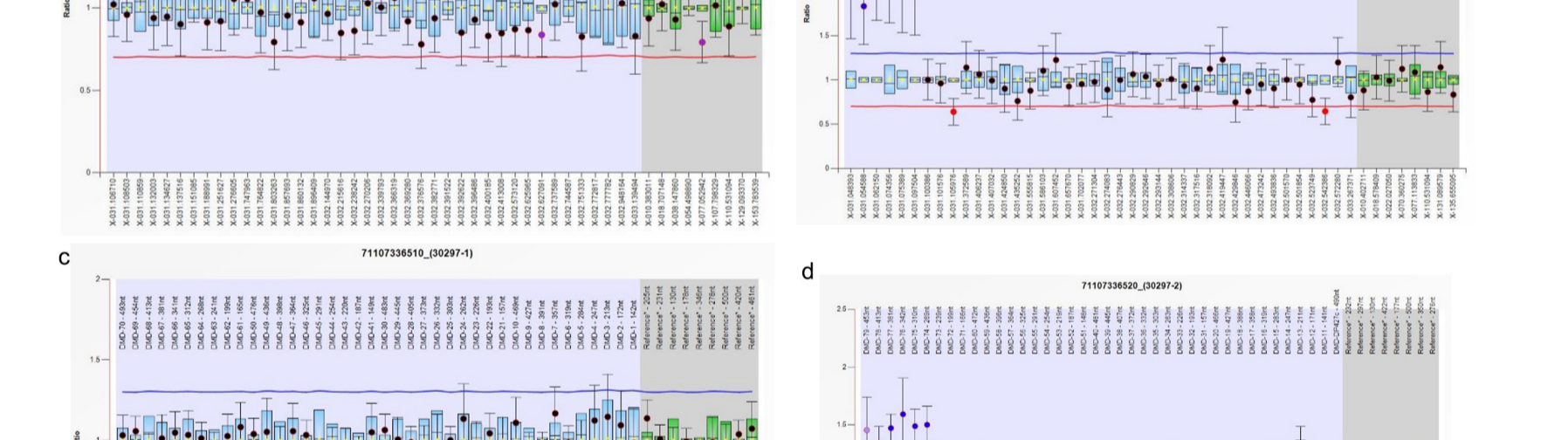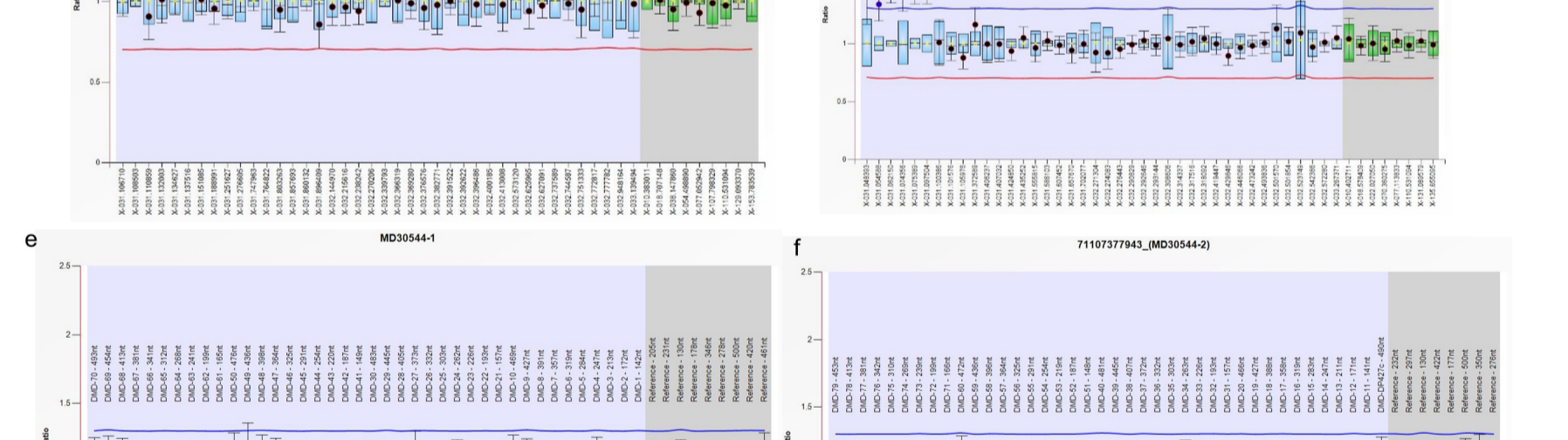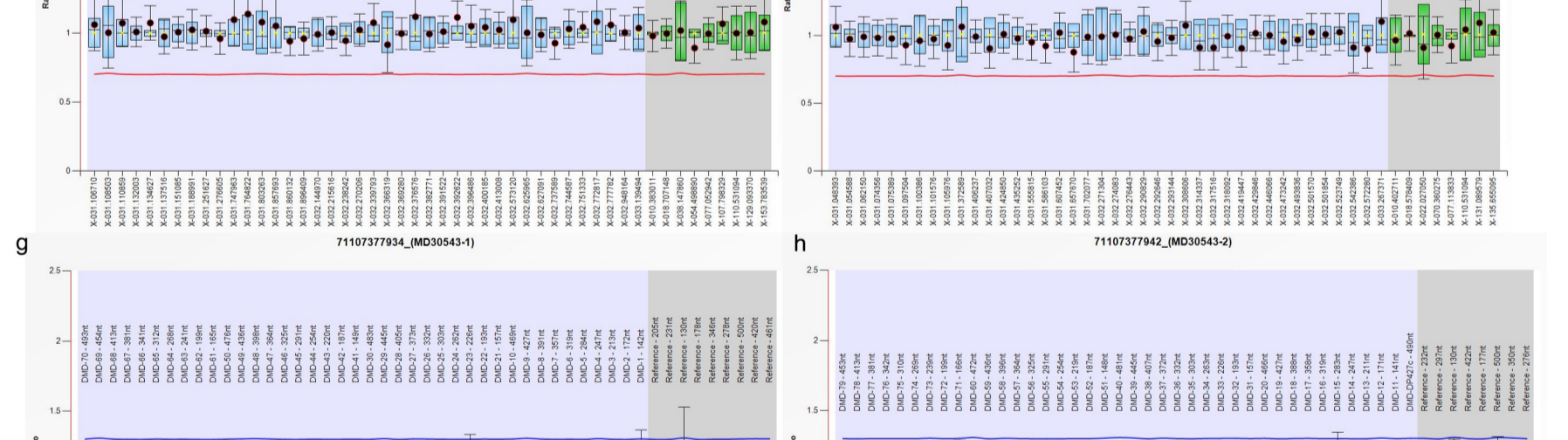

Supplement: Supplementary file 1 — Supplementary Material 1. [file 12920_2026_2333_MOESM1_ESM.pdf]
